# Effects of single- and multiple-dose oxytocin treatment on amygdala low-frequency BOLD fluctuations and BOLD spectral dynamics in autism

**DOI:** 10.1038/s41398-022-02158-8

**Published:** 2022-09-20

**Authors:** Kaat Alaerts, Sylvie Bernaerts, Nicole Wenderoth

**Affiliations:** 1 Department of Rehabilitation Sciences, Group Biomedical Sciences, Neuromodulation Laboratory, Neurorehabilitation Research Group, University of Leuven, KU Leuven, Leuven, Belgium; 2 Department of Health Sciences and Technology, Neural Control of Movement Lab, ETH Zurich, Zurich, Switzerland

**Keywords:** Autism spectrum disorders, Neuroscience

## Abstract

Prior neuroimaging clinical trials investigating the neural effects of intranasal administration of the neuropeptide oxytocin demonstrated a key role of the amygdala in oxytocin’s neuromodulatory effects. These studies mostly demonstrated the acute effects of single-dose administrations, examining task-dependent effects of oxytocin on brain activity elicited during explicit experimental tasks or stimuli presentations. The increased consideration of oxytocin as a potential ameliorating treatment in autism spectrum disorder (ASD) requires a better understanding of how multiple-dose oxytocin administration affects intrinsic, task-free, amygdala function. In this double-blind, randomized, placebo-controlled trial with between-subject design, 38 adult men with ASD underwent resting-state fMRI scanning before and after oxytocin or placebo treatment. Effects were assessed either after a single-dose administration, consisting of 24 international units, or after multiple-dose treatment, consisting of 4 weeks of once-daily nasal spray administrations. Compared to placebo, oxytocin induced a decrease in intrinsic resting-state BOLD signal amplitudes of the bilateral amygdala (fractional amplitudes of low-frequency fluctuations) and modulated cross-frequency interactions between adjacent BOLD frequency components. The right amygdala showed a pattern of reduced cross-frequency harmonicity, while the left amygdala showed a relative increase in harmonic cross-frequency interactions after oxytocin treatment. Notably, the direction and magnitude of BOLD spectral changes induced after a single-dose were qualitatively similar to treatment effects induced after multiple-dose treatment. Furthermore, the identified spectral changes in amygdalar BOLD amplitude and cross-frequency harmonicity were associated with improved feelings of tension, reflecting oxytocin’s anxiolytic, stress-reducing neuromodulatory role. The observed effects of oxytocin on amygdalar BOLD spectral characteristics and associated behaviors contribute to a deeper mechanistic understanding of the intrinsic, task-free neuromodulatory dynamics that underlie single- and multiple-dose oxytocin treatment in ASD. European Clinical Trial Registry (Eudract 2014-000586-45).

## Introduction

Over the past decade, scientific and lay interest in the neuropeptide oxytocin (OT) has increased considerably due to its putative neuromodulatory role in a wide range of complex social behaviors, including interpersonal bonding, affiliation, social attachment, and reduction of stress and anxiety (reviewed in refs. [1–4]). Related to its reported social and anxiolytic effects, intranasal administration of OT is increasingly considered as a possible treatment for distinct neuropsychiatric conditions, including autism spectrum disorders (ASD).

Endogenous OT is synthesized in the hypothalamus, where neurons of the paraventricular and magnocellular nuclei project to various areas of the central nervous system involved in complex (social) behaviors. Following preclinical research in animal models, researchers investigated the mechanisms underlying OT’s neuromodulatory function in the human brain using non-invasive brain imaging tools such as functional magnetic resonance imaging (fMRI). One key target that is identified to play a pivotal role in OT’s diverse psychosocial and behavioral effects are the amygdala regions, which receive direct axonal projections from hypothalamic nuclei and show a high density of OT receptors for direct bottom-up neuroendocrine modulation [5]. Recent meta-analytic analyses, including studies from both healthy and clinical populations broadly converged on the identification of attenuated amygdala responses after a single-dose of OT administration [6]. This observation is overall in line with preclinical findings of reduced amygdala reactivity through inhibitory GABAergic interneurons in rodent models [7]. A number of human fMRI studies also reported increased amygdala activation following OT administration and several factors relating to sex or psychopathology are put forward as potential sources of variability (reviewed in refs. [6, 8]). Note that also mechanistically, OT’s impact on human brain function is proposed to be dual, eliciting on the one hand, (i) an anxiolytic effect, reducing stress and anxiety, and on the other hand (ii) a social salience effect to facilitate attention to, and perception of social signals [9–11]. Since the amygdala form an integral part of the threat-processing circuit, as well as the social salience network, opposite directions of OT’s effect on amygdala activity are interpreted to reflect this dual action [11]. In other words, it is anticipated that variability in elicited effects in prior “task-dependent” neuroimaging trials is to a large extent also reflective of variation in methodological factors, including differential psychosocial task requirements or the nature of the presented stimuli [6, 12, 13].

Some initial studies explored OT’s effect on “intrinsic” resting-state neural activity which is important to elucidate neuromodulation by OT within the brain’s intrinsic functional neural architecture, unconstrained by explicit experimental tasks or stimuli. In most prior resting-state studies, treatment-induced changes in functional connectivity are explored by assessing correlations between the spontaneous, low-frequency fluctuations of the resting-state BOLD (blood-oxygenation-level-dependent) signal, thus allowing an investigation into the neuromodulatory effects of OT on the functional connections or *edges* between distinct *nodes* of the intrinsic neural architecture (for recent reviews of OT functional connectivity-fMRI (fc-fMRI) studies, see refs. [14] and [15]). To date, however, insights into the neuromodulatory effects of OT on *regional* or *nodal* neural function during the resting state remain sparse. Some studies demonstrated OT-induced changes in regional cerebral blood flow [16, 17]. Also, one prior study explored OT-induced changes in the fractional amplitude of low-frequency fluctuations (fALFF) of intrinsic BOLD signals, which, in contrast to fc-fMRI assessments, allows the identification of nodes with regionally altered activity in the resting brain [18]. Generally, a single-dose of OT induced a predominant pattern of reduced resting-state fALFF in healthy controls in various subcortical regions, including the thalamus, putamen, and insula [18]. In patients with chronic back pain, on the other hand, fALFF generally increased in the thalamus, caudate, and amygdala subcortical regions [18].

It is unclear whether these observations will extend to other patient populations, and more importantly, how intrinsic neural function is altered after multiple-dose treatment with OT (daily nasal spray administrations, over a course of consecutive days). Gaining a deeper mechanistic understanding into these neural substrates seems crucial considering that long-term OT treatment is increasingly considered as a potential novel treatment for several neuropsychiatric conditions. Especially in the field of autism spectrum disorder (ASD), an increasingly growing number of OT clinical trials are emerging reporting on the efficacy of OT treatment for symptom improvement. To date, however, an inconsistent pattern of behavioral results has emerged with some studies showing beneficial effects, while others identified no benefit of OT over placebo treatment (e.g., in children with ASD [19]) (see ref. [20] for an overview of recent multiple-dose OT clinical trials). To further our understanding of variability in (clinical) treatment responses, it is of great importance to gain deeper insights into the neural substrates that underlie OT treatment effects. To only a handful of pharmacological neuroimaging studies examined neural changes after multiple-dose OT administration in ASD. For example, in recent studies from our and other labs, multiple-dose OT treatment effects were demonstrated in terms of resting-state functional connectivity and task-related brain activity in distinct regions of the central oxytocinergic system (including the amygdala, superior temporal sulcus, anterior cingulate, and prefrontal regions) after a course of 6 or 4 weeks of OT treatment in individuals with ASD [21–23].

The current study aims to extend these prior reports, by examining—for the first time—the effects of both single- and multiple-dose OT treatment on intrinsic *nodal* neural function of the amygdala in young adult men with ASD. To do so, a double-blind, randomized, placebo-controlled trial with between-subject design was conducted in which resting-state fMRI scanning was performed before and after a single-dose (24 IU) as well as after a 4-week course of once-daily OT treatment. Importantly, regional fractional amplitudes of low-frequency BOLD signal fluctuations (fALFF) were assessed separately for two dominant BOLD oscillatory components to account for the heterogeneous temporal nature of the (spontaneous) neuronal activity. One component ranged from 0.05 to 0.1 Hz (BOLD1), the other from 0.1 to 0.17 Hz (BOLD2). Indeed, spectral analyses of BOLD signals identified clustered oscillatory activity over a broad range of frequencies, and it is anticipated that information regarding underlying neuronal activity is also carried by distinct components of the intrinsic BOLD frequency spectrum [24].

Further, in addition to the assessment of fALFF, also OT-induced changes in the BOLD components’ dominant, peak frequencies were assessed, as well as regional cross-frequency interactions between spectral components BOLD1 and BOLD2. While research into the contribution and cross-frequency interactions of BOLD spectral components is limited, prior EEG work provided converging evidence that cross-frequency interactions of neural oscillations form a central mechanism by which neural systems coordinate and integrate at different spatio-temporal scales [25, 26]. Within a novel theoretical account by Klimesch et al. [27–30], a specific frequency architecture of neural oscillations was put forward, suggesting that neural oscillations constitute a geometrical series of exponent 2 for optimally entailing frequency domains that allow cross-frequency coupling and decoupling. This notion is based on the mathematical fact that neural oscillations can only fully synchronize when their peak frequencies form harmonic 2:1 relationships, i.e., *f*_1_ = *f*_2_/2; with e.g., *f*_1_ = 0.07 Hz and *f*_2_ = 0.14 Hz. This arrangement allows a pattern of frequent and regular excitatory phase meetings (cross-frequency coupling). Non-harmonic cross-frequency relationships, on the other hand, based on the irrational golden mean 1.618..:1, provide the highest physiologically possible desynchronized state, reducing the occurrence of spurious, noisy, background coupling (*f*_1_ = *f*_2_/1.6; with e.g., *f*_1_ = 0.087 Hz and *f*_2_ = 0.14 Hz). This arrangement is therefore anticipated to characterize the resting state of the brain, in which no selective information processing takes place [31]. From EEG recordings, the specific arrangement of adjacent frequency bands generally concurs with a binary series of oscillatory rhythms, e.g., with *δ* = 2.5, *θ* = 5, *α* = 10, *β* = 20, *γ* = 40, and state- and task-dependent shifts in peak frequencies were shown to be associated with transient shifts in harmonic/non-harmonic cross-frequency dynamics [30, 32–36]. Also, in terms of the spectral variation of fMRI signals, cross-frequency interactions among different parts of the BOLD signal spectrum are expected to form an important mechanism for information transfer and coordination [29], i.e., by carrying different information regarding the underlying neuronal activity [37]. In other words, the intuitive notion of interpreting the neural frequency architecture in terms of a geometrically organized system of harmonic oscillators is anticipated to extend to the distinct oscillatory components observed within the BOLD frequency spectrum [29]. To date, however, its experimental application and functional relevance remain unexplored.

In view of that, the current study aimed at providing novel insights into the effect of single- and multiple-dose OT treatment on distinct spectral dynamics of the intrinsic resting-state BOLD signal recorded from bilateral amygdala. These assessments include calculations of BOLD amplitude, BOLD peak-frequency, and the formation of harmonic or non-harmonic cross-frequency relationships between peak frequencies of BOLD components 1 and 2, respectively, facilitating a state of coupling or decoupling between oscillatory BOLD rhythms.

Considering the lack of explicit experimental stimulation during resting state, both single- and multiple-dose OT treatments are primarily hypothesized to induce a reduction in intrinsic BOLD amplitudes (fALFF) within bilateral amygdala, reflective of OT’s anxiolytic, stress-reducing neuromodulatory effect. Also in terms of cross-frequency dynamics, OT administration was primarily hypothesized to facilitate the intrinsic neural frequency architecture within amygdala regions into a “decoupled” state characterizing a resting state with minimal regional cross-frequency information transfer. Accordingly, reduced harmonic 2:1 cross-frequency relationships and/or increased occurrence of non-harmonic 1.6:1 cross-frequency relationships were expected.

## Methods

### Participants

In this double-blind, randomized, placebo-controlled study with a parallel design, forty young adult men with a formal diagnosis of ASD were recruited to undergo resting-state fMRI scanning before and after OT or placebo (PL) treatment. Effects were assessed either after a single-dose administration or after multiple-dose treatment, consisting of 4 weeks of once-daily nasal spray administrations (see Supplementary Fig. 1 for the Consort Flow Diagram of participants in the trial).

Diagnosis of ASD was made by a multidisciplinary team based on the strict criteria of the DSM-IV-TR (Diagnostic and Statistical Manual of Mental Disorders). Prior to the study, the Autism Diagnostic Observation Schedule (ADOS) [38] and estimates of intelligence were acquired from all participants, using the 6-subtest short-version of the Wechsler Adult Intelligence Scale-IV—Dutch version (Table 1). Written informed consent was obtained from all participants prior to the study. Consent forms and study design were approved by the UZ/KU Leuven Ethics Committee for Biomedical Research (S56327) in accordance to The Code of Ethics of the World Medical Association (Declaration of Helsinki). The current report constitutes an exploratory investigation of changes in the fractional amplitude of low-frequency fluctuations (fAFF) and spectral dynamics of the resting-state BOLD signal recorded before and after nasal spray administration. The included resting-state fMRI data were adopted from a prior study [22] assessing conventional resting-state functional connectivity (registered at Eudract 2014-000586-45 and clinicaltrial.gov: NCT02940574).

**Table 1:** Demographic and clinical characteristics of participants randomized to receive oxytocin or placebo.

	Oxytocin	Placebo	*t*-value	*P*
	*N* = 21	*N* = 17		
Age	24.76 ± 4.85	24.06 ± 5.54	0.42	0.68
Handedness	16 R/5 L	15 R/2 L		
IQ				
Total IQ	101.76 ± 12.52	107.29 ± 18.91	−1.08	0.29
VIQ	105.57 ± 9.27	111.35 ± 12.99	−1.60	0.12
PIQ	104.76 ± 18.35	104.41 ± 21.88	0.05	0.96
ADOS				
Total	7.19 ± 4.312	7.59 ± 3.89	−0.29	0.77
Communication	2.14 ± 1.35	2.24 ± 1.44	−0.20	0.84
Social interaction	5.05 ± 3.41	5.35 ± 3.14	−0.28	0.78
RRB	1.19 ± 1.29	1.06 ± 0.9	0.36	0.72

			Pearson Chi-square	*P*
Use of psychostimulant medication*	5	2	0.91	0.34
Comorbidity*	7	2	2.42	0.12

*R* right, *L* left, *IQ* intelligence quotient, *VIQ* verbal IQ, *PIQ* performance IQ, *ADOS* autism diagnostic observation schedule, *RRB* restricted and repetitive behavior.

Mean ± standard deviation.

*Detailed information on medication use and comorbidities is provided in Supplementary Table 1.

### Nasal spray administration

Participants were randomized to receive OT or PL based on a computer-generated randomized order. All participants and research staff conducting the trial were blind to treatment allocation, except for the manager of randomization and masking of drug administration. OT (Syntocinon®, Sigma-tau) and placebo, consisting of saline natrium-chloride solution, were administered in amber 15 ml glass bottles with metered pump (ACA Pharma). All participants administered the first dose in front of the experimenter and received clear instructions about the use of the nasal spray in accordance with recommendations by [39]. During the 4-week treatment, participants self-administered a daily dose of 24 IU, using 3 puffs per nostril, over 4 consecutive weeks, rendering 28 doses in total. Participants were asked to administer the nasal spray in the morning and to keep a daily record of the time point of nasal spray administration. Overall, minimal side effects were noted, and compliance with the treatment was high (see ref. [40] for more detailed information).

### MRI data acquisition and analysis

Anatomical and resting-state fMRI images were acquired on a 3.0 Tesla Philips MR scanner (Best, The Netherlands) with an 8-channel phased-array head coil. Scanning was performed (i) at baseline; (ii) after a single-dose of nasal spray administration; and (iii) after the 4-week multiple-dose treatment. Single-dose fMRI scanning was performed ~45 min after nasal spray administration. The multiple-dose fMRI scanning was performed at least 24 h after the final administration to avoid residual, “acute” effects of the last dose. During the 7-min resting-state fMRI scans, participants were instructed to relax, but not sleep; to keep their eyes open while staring at a white cross and to think of nothing in particular. Detailed information on the scanning parameters, preprocessing, and head motion analysis is provided in Supplementary Methods and Supplementary Fig. 2. Note that since participants were recruited to participate in a larger clinical trial assessing the neural and behavioral effects of multiple-dose treatment with OT, the fMRI scanning protocol additionally included two other scan modalities (not part of the current report): (i) task-based fMRI scanning during biological motion processing [21] and (ii) diffusion tensor imaging; both performed after the acquisition of the resting-state scan.

#### Calculation of BOLD signal amplitude

For each participant, residual time series were averaged across all voxels in each left and right amygdala region-of-interest (ROI), as defined from the subcortical FSL Harvard-Oxford atlas. Time–frequency representations of the ROI BOLD time series were then obtained using Fast Fourier Transform (FFT) computed through the MATLAB *spectrogram* function (Hanning window length of 27 TR with 96.3% overlap, 0.001 Hz resolution between 0.04 and 0.2 Hz). Next, fractional amplitudes of the low-frequency fluctuations (fALFF, % of overall power) were estimated for two dominant BOLD oscillatory components, with frequency ranges from 0.05 to 0.1 Hz (BOLD1) and 0.1 to 0.17 Hz (BOLD2), corresponding to the BOLD intrinsic mode functions described in [24]. FALFF measures of each BOLD frequency component were obtained separately for each participant, ROI (amygdala right and left) and assessment session (baseline, single-dose, and multiple-dose).

#### Calculation of BOLD signal peak-frequency

In addition to the calculation of BOLD signal amplitudes, also transient BOLD signal peak frequencies were detected for each BOLD frequency band within each overlapping Hanning window using the find local maxima function, implemented in MATLAB r2020b (i.e., *findpeaks*) (similar to procedures described in refs. [32, 36]). With this algorithm, data samples larger than their two neighboring samples are identified as “local peaks” within the specified spectral frequency range of each BOLD component. For a limited number of windows, no clear peaks were detected, and these windows were excluded from further analyses (BOLD1: 8.87%; BOLD2: 2.11% of the total number of windows, across participants and sessions). When two or more peaks were detected in one frequency band, only the peak with the highest amplitude was selected. Mean peak frequencies of each frequency component were obtained separately for each participant, ROI, and session.

#### Calculation of regional BOLD harmonic and non-harmonic cross-frequency interactions

Cross-frequency harmonic and non-harmonic relationships between transient peak frequencies were assessed for the left and right amygdala ROI. To do so, the numerical ratio of the BOLD1 and BOLD2 peaks (peak-frequency_BOLD2_/ peak-frequency_BOLD1_) was calculated for each window and rounded to the first decimal place (e.g., 0.14/0.07 Hz = 2.0). Hence, the obtained ratio values ranged between 1.1 and 3.2 in steps of 0.1. Next, the proportion of windows in which the BOLD2:BOLD1 peak ratio equaled 1.6 or 2.0, respectively, representing non-harmonic and harmonic relationships, was determined for each participant, ROI, and session.

**Fig. 1:**
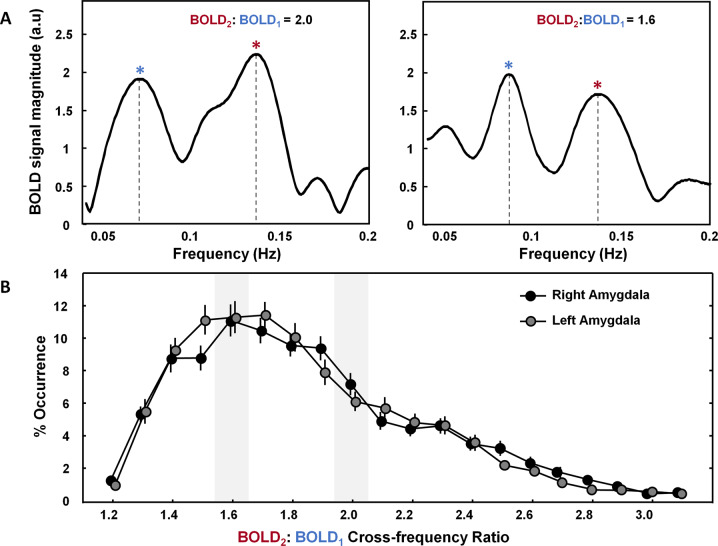
Transient detection of BOLD1 and BOLD2 peak frequencies and determination of cross-frequency relationships. Peak frequencies of BOLD oscillatory components, with frequency ranges 0.05–0.1 Hz (BOLD1) and 0.1–0.17 Hz (BOLD2) were detected transiently, and the numerical ratio between the BOLD2 and BOLD1 peak frequencies was calculated (peak-frequency_BOLD2_/peak-frequency_BOLD2_). **A** Visualization of the BOLD frequency spectra of two exemplary windows in which the identified BOLD2 and BOLD1 peak frequencies formed a “harmonic” (2:1) versus a “non-harmonic” (1.6:1) cross-frequency relationship. **B** Visualization of the relative occurrence of all possible cross-frequency relationships (% occurrence) as recorded at baseline (before nasal-spray administration), showing a maximal occurrence of the non-harmonic 1.6:1 BOLD2:BOLD1 ratio aspect both in the right and left amygdala (occurrence of respectively, 11.3% and 11.1%, average across participants). The harmonic 2:1 ratio aspect showed an average occurrence of 4.7% in the right and 5.8% in the left amygdala region.

Figure 1A visualizes the FFT frequency spectra of two exemplary windows in which the identified BOLD2 and BOLD1 peak frequencies formed a “harmonic” (2:1) versus a “non-harmonic” (1.6:1) cross-frequency relationship. Figure 1B visualizes the distribution of the percentage occurrence of all possible ratios, as recorded at the baseline session, before nasal spray administration. Overall, the distribution showed a maximal occurrence of the non-harmonic 1.6:1 BOLD2:BOLD1 ratio aspect, both in the right and left amygdala, indicating an occurrence of respectively, 11.3% and 11.1. Accordingly, this configuration formed the most prominent physiological state within the intrinsic BOLD frequency architecture. The harmonic 2:1 ratio aspect showed an average occurrence of 4.7% in the right and 5.8% in the left amygdala region.

### Statistical analysis

#### Treatment-related effects

To explore treatment-related changes in the obtained spectral parameters, changes from baseline were calculated for each parameter (i.e., fALFF, BOLD peak-frequency, cross-frequency harmonicity/non-harmonicity). Next, the change-from-baseline (baseline-subtracted) scores were subjected to repeated-measures ANOVA analyses with the between-subject factor “treatment” (OT, PL) and the within-subject factors “assessment session” (single-dose, multiple-dose), “hemisphere” (right, left amygdala) and “BOLD frequency component” (BOLD1, BOLD2). Note that for all variables, baseline scores (assessed before allocated treatment) were not significantly different between groups (all, *t* < 1.7; *P* > 0.05).

#### Brain-behavior relationship

As outlined in more detail in ref. [41], treatment-related changes in self-reported mood were assessed previously using the Profile of Mood States (POMS). This scale consists of five subscales, each rated on a five-point Likert, i.e., tension, depression, vigor, fatigue, and anger [42]. Interestingly, participants receiving the 4-week course of OT treatment reported higher feelings of “vigor” (feeling “energetic”, “active”, “lively”), compared to PL. Both groups also reported improvements in feelings of tension and fatigue, but for these subscales, the effects were not treatment-specific [41]. Here, we re-used the behavioral data of these subscales for assessing brain-behavior relationships using step-wise multiple-regression models (forward selection), with the neural spectral variables (recorded after the multiple-dose treatment) as a dependent variable and the POMS subscales (vigor, tension, fatigue), as well as the factor “treatment” (OT, PL) as predictor variables.

All statistics were performed with Statistica 14 (Tibco Software Inc.).

## Results

### Effect of single- and multiple-dose OT treatment on amygdala BOLD signal amplitude

**Fig. 2:**
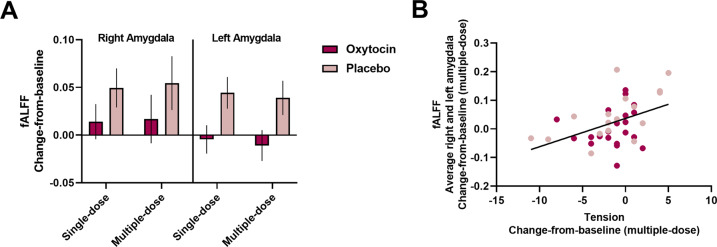
Effect of single- and multiple-dose oxytocin treatment on amygdala BOLD signal amplitude. **A** Mean changes-from-baseline in the fractional amplitude of low-frequency fluctuations (fALFF) are visualized separately for each treatment group (oxytocin, placebo), assessment session (single-dose, multiple-dose), and amygdala region (right, left). Data are visualized and averaged across BOLD components 1 and 2. Both after a single-dose and after multiple-dose treatment, the oxytocin group displayed significantly lower amygdalar fALFF, compared to the placebo group. Vertical bars denote ± standard errors. **B** Visualization of the brain-behavior relationship between multiple-dose changes-from-baseline in self-reported feelings of tension (assessed with the Profile of Mood Scale) and multiple-dose changes-from-baseline in fALFF (averaged across amygdala regions and BOLD components).

ANOVA analyses showed a main effect of “treatment”, indicating that change-from-baseline scores in amygdala low-frequency BOLD fluctuations (fALFF) were significantly lower in the OT group, compared to the placebo group (*F*(1,36) = 5.42; *P* = 0.026; *ŋ*^*2*^ = 0.13) (Fig. 2A). No significant “treatment × assessment session” interaction was revealed (*F*(1,36) = 0.01; *P* = 0.94; *ŋ*^*2*^ < 0.001), indicating that changes-from-baseline in fALFF scores were significantly lower after the OT treatment, both acutely, after the single-dose administration, as well as chronically, after the multiple-dose OT treatment. None of the other main or interaction effects reached significance (all *P* > 0.05), indicating that the effect was evident irrespective of the amygdala region or BOLD frequency component.

Step-wise multiple-regression analysis with forward selection (*F* > 1.00) rendered a significant model, retaining the subscale “tension” (*β* = 0.43; *t*(35) = 3.04; *P* = 0.005; Pearson *r* = 0.44; *P* = 0.006) and the factor “treatment” (*β* = 0.29; *t*(35) = 1.99; *P* = 0.05) as significant predictors of pre-to-post changes in fALFF after the multiple-dose treatment, explaining 27% of the variance (*R*^*2*^ = 0.27; *F*(2,35) = 6.61; *P* < 0.004). The relationship with tension implied that individuals who displayed more improvements in self-reported feelings of tension also displayed more reduced fALFF after the multiple-dose treatment (Fig. 2B).

### Effect of single- and multiple-dose OT treatment on BOLD peak frequencies and regional cross-frequency interactions

**Fig. 3:**
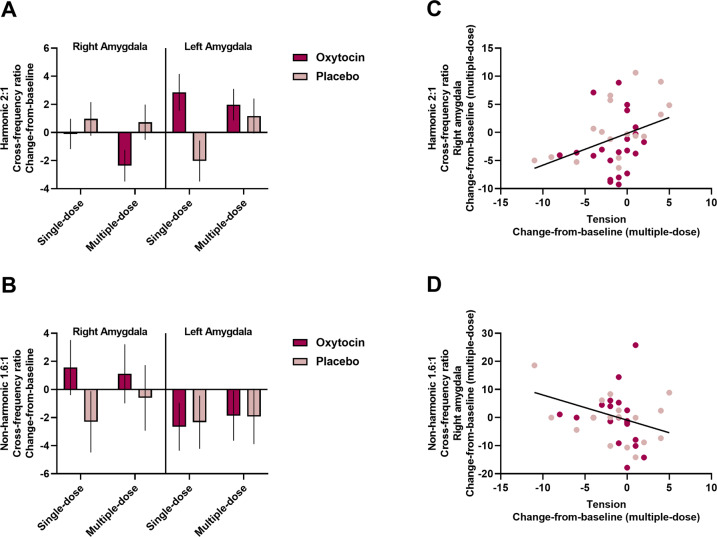
Effect of single- and multiple-dose oxytocin treatment on amygdala BOLD cross-frequency interactions. **A** Mean changes-from-baseline in the transient occurrence of the harmonic 2:1 cross-frequency ratio aspect (% occurrence) are visualized separately for each treatment group (oxytocin, placebo), assessment session (single-dose, multiple-dose), and amygdala region (right, left). Both after a single-dose and after multiple-dose treatment, the oxytocin group displayed a reduction in regional cross-frequency harmonicity in the right amygdala and an increase in harmonicity in the left amygdala. **B** Mean changes-from-baseline changes in the transient occurrence of the non-harmonic 1.6:1 cross-frequency ratio aspect (% occurrence) are visualized separately for each treatment group (oxytocin, placebo), assessment session (single-dose, multiple-dose) and amygdala region (right, left). Vertical bars denote ± standard errors. **C** Visualization of the brain-behavior relationship between multiple-dose changes from baseline in self-reported feelings of tension (assessed with the Profile of Mood Scale) and multiple-dose changes from baseline in the transient occurrence of the harmonic 2:1 cross-frequency ratio aspect in the right amygdala. **D** Visualization of the relationship between multiple-dose changes from baseline in self-reported feelings of tension and multiple-dose changes from baseline in the transient occurrence of the non-harmonic 1.6:1 cross-frequency ratio aspect in the right amygdala.

ANOVA analyses of 2:1 harmonic cross-frequency interactions (% transient occurrence of 2:1 harmonic BOLD2:BOLD1 cross-frequency ratio) showed a “treatment × region’ interaction effect (*F*(1,36) = 7.96; *P* = 0.008; *ŋ*^*2*^ = 0.18). The interaction effect indicated reduced change-from-baseline scores in cross-frequency harmonicity in the right amygdala and higher change-from-baseline scores in harmonicity in the left amygdala in the OT group, compared to the placebo group. No interaction with the factor ‘assessment session’ was revealed (all *P* > 0.05), indicating that the effect was evident, both after the single-dose administration, as well as after the multiple-dose treatment (Fig. 3A). The incidence of non-harmonic cross-frequency interactions (% transient occurrence of 1.6:1 non-harmonic BOLD2:BOLD1 cross-frequency ratio) was not significantly modulated by single- or multiple-dose OT treatment (all, *P* > 0.05) (Fig. 3B). Also no treatment-related changes were evident in terms of BOLD1 or BOLD2 peak frequencies (all *P* > 0.05) (Supplementary Fig. 3). These observations indicate that the OT treatment did not induce overall accelerations or decelerations of BOLD peak-frequency per se, but rather modulated the incidence of cross-frequency harmonicity between the respective BOLD frequency components.

In terms of the observed pre-to-post *decreases* in 2:1 regional cross-frequency harmonicity of right amygdala, step-wise multiple-regression analysis (*F* > 1.00) rendered a significant model retaining the subscale “tension” (*β* = 0.36; *t*(35) = 2.41; *P* = 0.021; *r* = 0.36; *P* = 0.026) and the factor “treatment” (*β* = 0.29; *t*(35) = 1.95; *P* = 0.059), explaining 22% of the variance (*R*^*2*^ = 0.22; *F*(2,35) = 4.82; *P* < 0.014). The relationship with tension implied that individuals who displayed more improvements in self-reported feelings of tension also displayed more reductions in right amygdala cross-frequency harmonicity (Fig. 3C).

In terms of 1.6:1 non-harmonicity of right amygdala, an opposite pattern was revealed, indicating that individuals who displayed more improvements in self-reported feelings of tension displayed stronger increases in non-harmonic cross-frequency interactions (model: *R*^*2*^ = 0.10; *F*(1,36) = 4.10; *P* < 0.05; tension: *β* = −0.32; *t*(36) = −2.04; *P* = 0.05; *r* = −0.32; *P* = 0.05, Fig. 3D).

For changes in 1.6:1 non-harmonicity of left amygdala, the subscales “fatigue” and “tension” were retained in the regression model (model: *R*^*2*^ = 0.17; *F*(2,35) = 3.58; *P* < 0.039) indicating that higher left amygdala non-harmonicity was associated with increased feelings of fatigue (*β* = 0.45; *t*(35) = 2.61; *P* = 0.013; *r* = 0.33; *P* = 0.047) and trend-level reductions in tension (*β* = −0.28; *t*(35) = −1.65; *P* = 0.11). Step-wise multiple regression with changes in left amygdala 2:1 cross-frequency harmonicity as dependent variable retained no significant predictor variables (*R*^*2*^ = 0.05; *F*(1,36) = 1.89; *P* < 0.18).

In short, at the group level, individuals receiving the OT treatment showed overall reduced cross-frequency harmonicity between BOLD spectral components 1 and 2, especially in the right amygdala. Between-subject dimensional analyses showed that changes in this neural spectral parameter were associated with improved feelings of tension.

## Discussion

The current study revealed significant effects of OT treatment on distinct spectral dynamics of the intrinsic resting-state BOLD signal recorded in the bilateral amygdala, indicating lower BOLD signal amplitude (fALFF) after OT, compared to PL treatment, irrespective of BOLD frequency range. The identification of attenuated regional “intrinsic” neural function within bilateral amygdala during resting-state forms an important extension of prior neuroimaging OT pharmacological trials demonstrating either increases or decreases in task-related amygdalar activity, depending on experimental task design/stimuli or other (person-dependent) factors [6, 12, 13]. Our observations of single- and multiple-dose OT-induced modulations of fALFF also extend findings of a previous single-dose administration trial, demonstrating, in healthy controls, a predominant pattern of reduced resting-state fALFF in various subcortical regions [18]. In patients with chronic back pain, however, opposite effects were evident, indicating OT-induced increases in fALFF, particularly for patients with higher symptom load (i.e., larger baseline scores in pain vigilance and awareness/fear of pain) [18]. In the current study, it appeared that pre-to-post increases in amygdalar fALFF were mostly evident in participants of the PL group, whereas after OT treatment, these increases were absent or largely dampened. In a previous report from our lab, the effects of multiple-dose OT treatment on task-based amygdala activity during processing of body language from point-light displays were examined, similarly showing an overall pattern of dampened task-dependent amygdala reactivity [21]. Also, in our prior report examining conventional resting-state functional connectivity, a predominant pattern of reduced functional connectivity of the amygdala was revealed (mostly to prefrontal and superior temporal regions) after multiple-dose OT treatment [22]. Together, these reports indicate that, aside from task-dependent effects, OT-induced modulations are also evident for intrinsic neural function (resting-state BOLD amplitude), both after a single-dose or after multiple-dose OT treatment.

Aside from BOLD amplitude, the current study also explored OT treatment effects on other spectral dynamics, including BOLD peak-frequency, and the formation of harmonic or non-harmonic cross-frequency relationships between peak frequencies of BOLD components 1 and 2. While no overall accelerations or decelerations in dominant peak frequencies were observed, OT modulated the incidence of cross-frequency harmonicity between the respective BOLD frequency components, indicating a pattern of reduced cross-frequency harmonicity in the right amygdala, while the left amygdala showed a relative increase in harmonic cross-frequency interactions after OT treatment. Notably, the direction and magnitude of BOLD spectral changes were similar after a single-dose administration or after the multiple-dose treatment, indicating that the priming of amygdala neural substrates is qualitatively similar after a single-dose or after prolonged use of oxytocin over the 4-week period. Furthermore, considering the identified associations with improved feelings of tension, the observed effects are assumed to be indicative of OT’s anxiolytic, stress-reducing neuromodulatory role [43]. Indeed, current models of amygdala-mediated anxiolytic effects suggest that OT may predominantly activate oxytocinergic neurons in the lateral part of the central amygdala, which, through GABAergic projections, exerts inhibitory control over the medial central amygdala thereby impacting the associated top–down control of anxiety and fear responses [44, 45]. Further, the observation that brain-behavior relationships with improved feelings of tension were predominantly observed for OT-induced changes in right amygdala (not left), is likely reflective of the predominant role of right amygdala in aversive behavior and negative emotions [43].

Treatment-related effects on cross-frequency harmonicity were only significantly evident in terms of a modulation of the incidence of 2:1 harmonic cross-frequency relationships, whereas the incidence of 1.6:1 non-harmonic cross-frequency interactions remained relatively unchanged, despite the observation that this configuration forms a highly prevalent physiological state within the intrinsic BOLD frequency architecture. Albeit speculative, the naturally high occurrence of the 1.6:1 ratio aspect could indicate that its occurrence constitutes a “trait” aspect of the resting brain (as suggested before in [31]), perhaps rendering it less susceptible to “state-dependent” modifications, as induced by OT treatment. Nonetheless, irrespective of treatment, changes in both ratio aspects were inversely associated with behavioral changes, such that increases in the 1.6:1 non-harmonic and concurrent decreases in the 2:1 harmonic ratio aspect were associated with improved self-reported feelings of tension, particularly for right amygdala. For left amygdala, also an association between increased feelings of fatigue and a higher incidence of 1.6:1 non-harmonicity was evident. Together, these observations largely support the purported notion that cross-frequency *de*coupling constitutes an important neural correlate of the resting brain in a task-free, restful state [31].

Prior EEG work showed the occurrence of 2:1 harmonic cross-frequency arrangements between EEG alpha and theta oscillatory rhythms to be most prominent during active cognitive processing, whereas the formation of 1.6:1 non-harmonic arrangements were more prominent during rest and a non-cognitively demanding breath focus meditation conditions [32, 33, 35]. Also, in relation to OT, a recent single-dose administration study showed a relative increase in the transient formation of alpha–theta 1.6:1 non-harmonic cross-frequency configurations, whereas the occurrence of 2:1 harmonic cross-frequency arrangements was relatively decreased after a single dose of OT [36]. Notably, in the latter study, baseline changes in cross-frequency relationships were paralleled by increased parasympathetic drive, as indexed using heart rate variability. This observation therefore demonstrated a link between neural *de*coupling and increased parasympathetic tone. While it is difficult to draw direct comparisons between these EEG-based observations and the current BOLD cross-frequency dynamics, it is speculated that the identified changes in amplitude and cross-frequency harmonicity in BOLD spectral frequencies constitute an important neural mechanism by which OT administration may promote a resting state with minimal regional cross-frequency information transfer i.e., for facilitating homeostatic balance.

Within distinct lines of work, the induction of high signal-to-noise states has been suggested to form a shared over-arching mechanism by which OT may exert its widespread neuromodulatory function [46]. For example, at the neuronal level, OT enhances signal-to-noise ratio properties of synaptic transmissions by suppressing spontaneous (noisy) neuronal firings thereby enhancing the fidelity of spike transmission [46]. In vivo rodent research demonstrated OT receptor activity of olfactory bulb neurons to increase peak firing responses to social sensory information (odorants) by lowering their spontaneous baseline firing, hence augmenting signal-to-noise properties for signal transmission [47]. Also, in recent human work, OT is suggested to act as a neuromodulatory filter, favoring the processing of “salient” survival-relevant information by dampening irrelevant background noise [36, 48, 49]. In this view, the overall amplitude attenuation and minimization of cross-frequency interactions within amygdalar BOLD signals during inactive rest may not be solely reflective of OT’s stress-reducing role. It may also form an important mechanism by which OT optimally prepares the system for forthcoming active signal processing, specifically by optimally reducing residual “background” amygdala activity when explicit external stimulation is absent.

The following limitations and directions for future research are noted. First, the current study included only young adult men with ASD, rendering the generalizability of the observed effects to women, or other patient populations uncertain. Further, in the current study, participants administered the OT nasal spray once a day in the morning (similar to ref. [50] while the majority of prior multiple-dose OT studies administered two doses/day, one in the morning and one in the afternoon (e.g., ref. [23]). Interestingly, recent work showed that intermittent, every other day, multiple-dose administration may be therapeutically most efficient for reducing amygdala reactivity and inducing anxiolytic behavioral effects [51, 52]. In light of the current observations of multiple-dose neural effects of OT treatment, future trials should be directed at identifying the optimal dosing, administration length, and intervals.

To conclude, this study revealed significant effects of OT treatment on distinct spectral dynamics of the intrinsic resting-state BOLD signal recorded in the bilateral amygdala. Overall, the direction and magnitude of BOLD spectral changes were qualitatively similar after a single- or multiple-dose treatment and indicative of OT’s anxiolytic, stress-reducing neuromodulatory effects.

## Supplementary information

Supplementary Material
