# Supplementary material for: Effects of single- and multiple-dose oxytocin treatment on amygdala low-frequency BOLD fluctuations and BOLD spectral dynamics in autism
Source: Transl Psychiatry. 2022 Sep 20;12:393. doi: 10.1038/s41398-022-02158-8 (PMC9489696; doi:10.1038/s41398-022-02158-8)
Supplement: Supplementary file 1 — Supplementary Material [file 41398_2022_2158_MOESM1_ESM.pdf]

Supplementary Materials

Effects of single- and multiple-dose oxytocin treatment on amygdala low-frequency BOLD fluctuations and BOLD spectral dynamics in autism

Kaat Alaerts, Sylvie Bernaerts, Nicole Wenderoth

Supplementary Methods

MRI scanning parameters. Anatomical imaging consisted of a high-resolution structural volume acquired using a coronal three-dimensional turbo field echo T1-weighted sequence with the following parameters: 182 contiguous coronal slices covering the whole brain and brainstem, slice thickness = 1.2 mm; repetition time (TR) = 9.4 ms; echo time (TE) = 4.6 ms; matrix size = 208 x 207; field-of-view (FOV) = 250 x 250 mm; in-plane pixel size = 1.2 x 1.2 mm²; acquisition time = 1 min 43 s. Resting-state fMRI images were acquired using a T2*-weighted gradient-echo echo planar imaging (GE-EPI) sequence with the following parameters: TR = 2500 ms; TE = 30 ms; matrix size = 80 x 78, FOV = 200 x 200 mm; flip angle 90°; slice thickness = 2.7 mm, slice gap = 0.4 mm; axial slices = 45; 162 functional volumes; acquisition time = 7 min.

MRI data preprocessing. The CONN functional connectivity toolbox 16.b (5) was used for image preprocessing implemented in Matlab R2020b (Mathworks). Resting-state fMRI images were spatially realigned, normalized to the standard EPI-template of the Montreal Neurological Institute (MNI-152) and resampled into 3-mm isotropic voxels. Realignment parameters were modeled as regressors of no-interest and white matter and cerebrospinal fluid were removed as confounds following the implemented CompCor-strategy in the CONN toolbox (Behzadi et al., 2007). To assess potential influences of head motion, frame-wise displacement was computed for each scan at each session. However, no group- (OT, PL) or session-related differences were identified (**Supplementary Figure 2**)

Supplementary Figure 1.

CONSORT Flow Diagram.

MRI scanning was performed at baseline; after a single dose of oxytocin or placebo treatment and after a four-week course of oxytocin/ placebo treatment. One participant of the oxytocin group was excluded from the analyses, due to self-termination of participation (unrelated to the treatment). One participant of the placebo group was excluded due to excessive in-scanner head motion (98.5% of images with framewise displacement exceeding >0.5 mm).

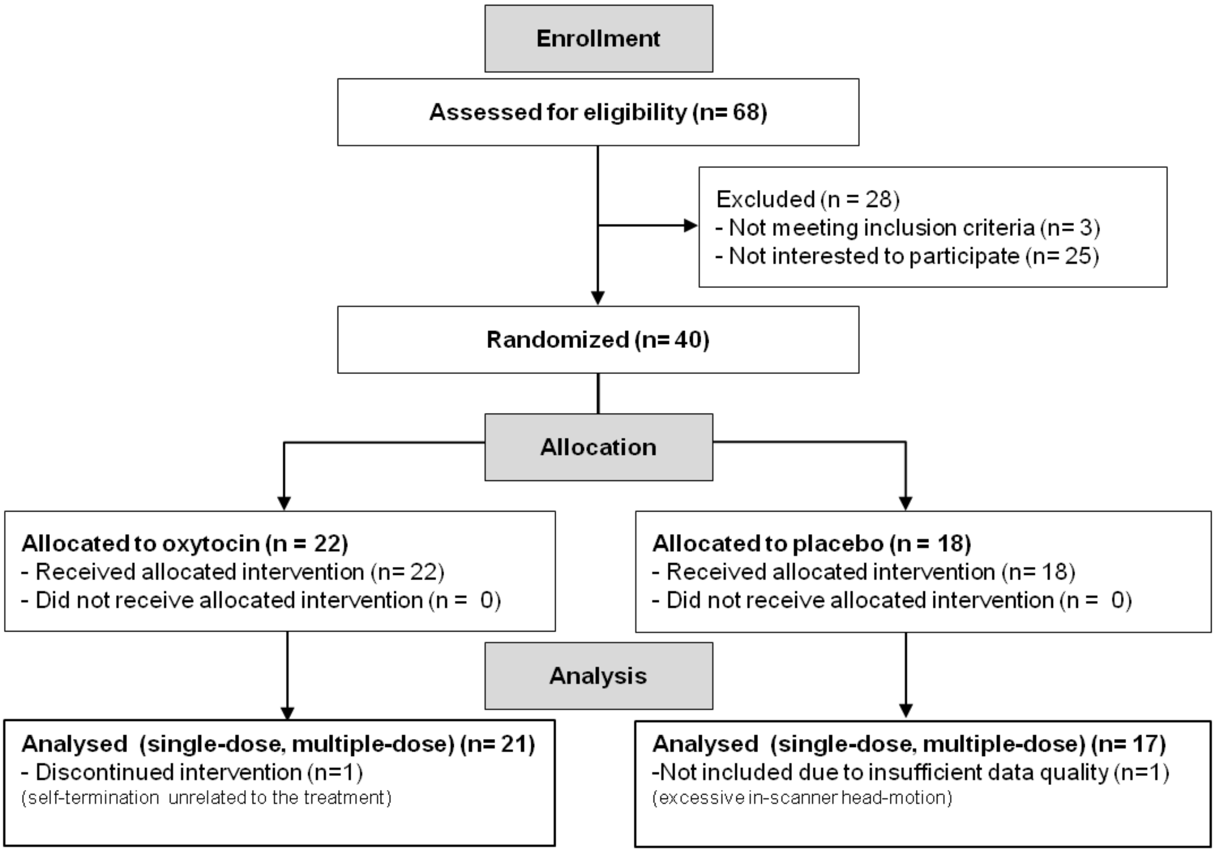

Supplementary Figure 2

Head motion analysis of the resting-state fMRI scans.

For all participants, head motion (mean frame-wise displacement (mean FD)) of the resting-state fMRI scans was assessed before (pre) and after the single-dose (post acute) and multiple-dose (post chronic) nasal spray administration. Across sessions, mean FD scores were not significantly different between treatment groups ($F(1, 36)= .17$; $p= .68$). Also no significant effect of 'session' ($F(2, 72)= .21$; $p= .81$) or 'session x treatment' interaction ($F(2, 72)=2.88$; $p= .062$) was revealed.

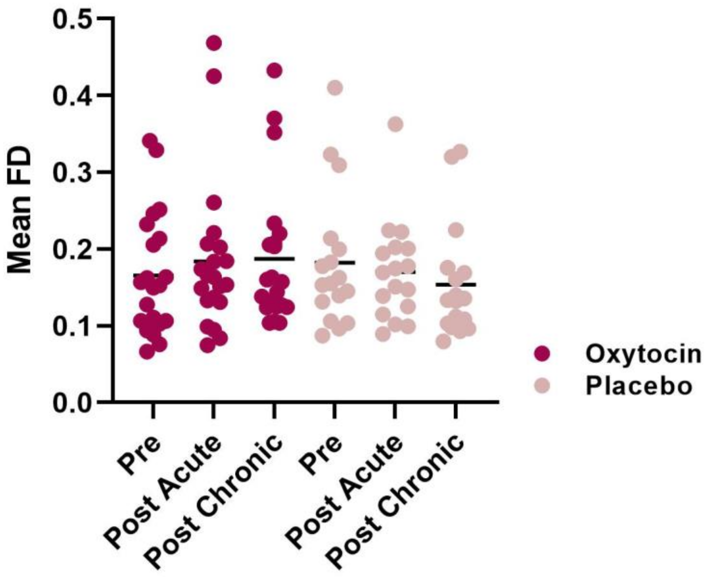

Supplementary Figure 3

Effect of single- and multiple-dose oxytocin treatment on amygdala BOLD peak frequencies

For each BOLD frequency component (**panel A**: BOLD1 (0.05 to 0.1 Hz); **panel B**: BOLD 2 (0.1 to 0.17 Hz)), mean changes-from-baseline in peak frequencies are visualized separately for each treatment group (oxytocin, placebo), assessment session (single-dose, multiple-dose) and amygdala region (right, left). Vertical bars denote ± standard errors.

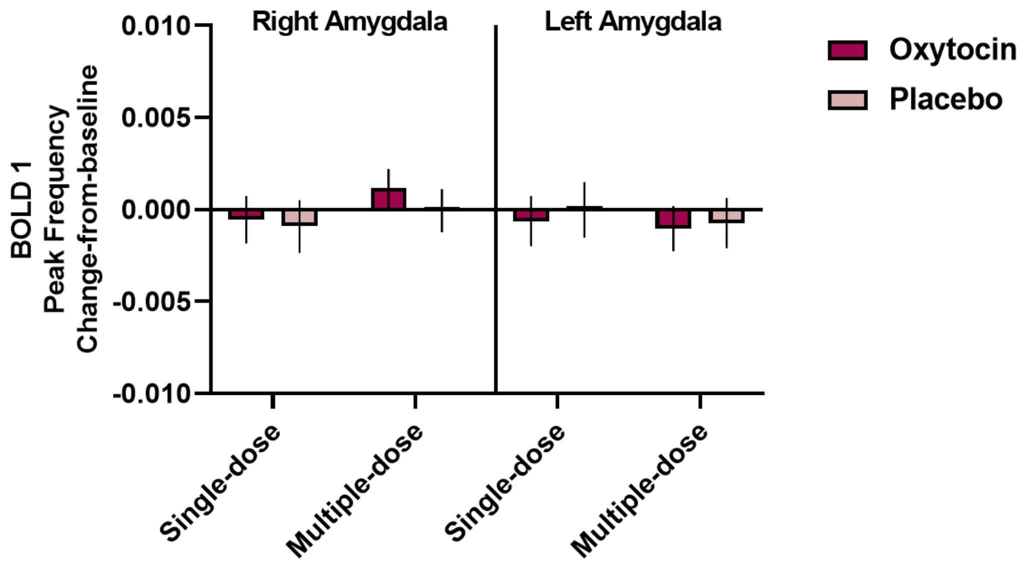

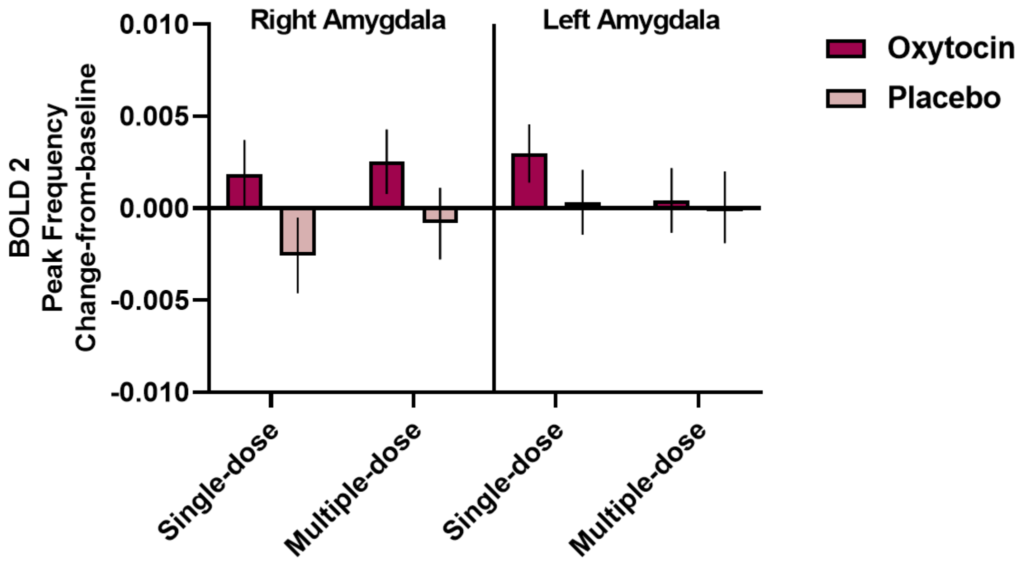

Supplementary Table 1

Detailed information on comorbidities and medication use for participants of the oxytocin and placebo group.

Comorbidities were screened through self-report (with the explicit mentioning of examples in the screening interview including e.g., ADHD, depression, dyscalculia, dyslexia). Current psychoactive medication use was defined as use within three months before study enrollment.

		Comorbidities	Medication use
**Oxytocin group**		**N= 7**	**N= 5**
	**i**	ADHD	Abilify, Tegretol
	**ii**	Depression	Welbutrine XR, Leviron, Cymbalta
	**iii**	Depression, ADD	Trazodone Mylan, Medikinet
	**iv**	Bipolar disorder	Maniprex, Bellozal, Mometasone
	**v**	ADHD,Dyslexia	/
	**vi**	ADHD, Depression	/
	**vii**	Dyslexia	/
	**viii**	/	Risperdal, Venlafaxine
**Placebo group**		**N= 2**	**N= 2**
	**i**	ADHD	/
	**ii**	ADHD	/
	**iii**	/	Zolpidem, Remergon, Rilatine
	**iv**	/	Trazodone, Escitalopram

ADHD: attention deficit hyperactivity disorder; ADD: attention deficit disorder
